# Antileishmanial Effect of 5,3′-Hydroxy-7,4′-dimethoxyflavanone of *Picramnia gracilis* Tul. (Picramniaceae) Fruit: *In Vitro* and *In Vivo* Studies

**DOI:** 10.1155/2015/978379

**Published:** 2015-04-30

**Authors:** Sara M. Robledo, Wilson Cardona, Karen Ligardo, Jéssica Henao, Natalia Arbeláez, Andrés Montoya, Fernando Alzate, Juan M. Pérez, Victor Arango, Iván D. Vélez, Jairo Sáez

**Affiliations:** ^1^PECET, Medical Research Institute, School of Medicine, University of Antioquia (UdeA), Calle 70, No. 52-21, A.A. 1226, Medellín, Colombia; ^2^Chemistry of Colombian Plants, Institute of Chemistry, Exact and Natural Sciences School, University of Antioquia (UdeA), Calle 70, No. 52-21, A.A. 1226, Medellín, Colombia; ^3^Botanical Studies, Institute of Biology, Exact and Natural Sciences School, University of Antioquia (UdeA), Calle 70, No. 52-21, A.A. 1226, Medellín, Colombia; ^4^Pharmacy School, University of Antioquia (UdeA), Calle 70, No. 52-21, A.A. 1226, Medellín, Colombia

## Abstract

Species of* Picramnia* genus are used in folk medicine to treat or prevent skin disorders, but only few species have been studied for biological activity and chemical composition.* P. gracilis* Tul. is a native species from Central and South America and although its fruits are edible, phytochemical analysis or medicinal uses of this species are not known. In the search of candidates to antileishmanial drugs, this work aimed to evaluate the antileishmanial activity of* P. gracilis* Tul. in* in vitro* and* in vivo* studies. Only ethanolic extract of fruits showed leishmanicidal activity. The majoritarian metabolite was* 5,3*′*-hydroxy-7,4*′*-dimethoxyflavanone* ether that exhibited high activity against* L. (V.) panamensis* (EC_50_ 17.0 + 2.8 mg/mL, 53.7 *μ*M) and low toxicity on mammalian U-937 cells, with an index of selectivity >11.8.* In vivo* studies showed that the flavanone administered in solution (2 mg/kg/day) or cream (2%) induces clinical improvement and no toxicity in hamsters with CL. In conclusion, this is the first report about isolation of* 5,3*′*-hydroxy-7,4*′*-dimethoxyflavanone* of* P. gracilis* Tul. The leishmanicidal activity attributed to this flavanone is also reported for the first time. Finally, the* in vitro* and* in vivo* leishmanicidal activity reported here for* 5,3*′*-hydroxy-7,4*′*-dimethoxyflavanone* offers a greater prospect towards antileishmanial drug discovery and development.

## 1. Introduction

Leishmaniasis is a tropical disease caused by* Leishmania* parasites that affects about 12 million people in 99 countries. Approximately, 350 million people are at risk of infection and two million new cases occur yearly [1]. Despite high morbidity, therapeutic alternatives are very few and have serious drawbacks associated mainly with use of high doses and prolonged administration resulting in moderate to severe toxicity [2]. The presence of severe toxic reactions to conventional medication indicates the need for new therapies that cure leishmaniasis. Natural products of plant origin are potential tools for these discoveries and have been used for centuries to treat empirically parasitic diseases for people around the world, stimulating clinical and laboratory research [3].

*Picramnia* species (Picramniaceae, previously Simaroubaceae) are commonly used in folk medicine to treat or prevent dermatosis, external ulcers (sores), and skin irritations [4]. Phytochemical investigation in some of these* Picramnia* species resulted in isolation of several metabolites [5, 6], mainly triterpenes [7–9], anthrones and anthraquinone glycosides [10–14], and oxanthrones [10–12, 15] with cytotoxic, antimicrobial, antifungal, or antiparasitic activities [16–18].* Picramnia gracilis* Tul. is a native species of the Andean region in South and Central America [19]. Although fruits of* P. gracilis* Tul. are edible [20], reports about phytochemical analysis or medicinal uses of this species are not known. Moreover, there are no reports about antileishmanial activity of any of* Picramnia* species.

Motivated by the presence of metabolites with antiparasitic activity previously demonstrated in several* Picramnia* species and their traditional use in skin problems, this study aimed to discover antileishmanial activity and cytotoxicity in extracts and metabolites of leaves and fruits of* P. gracilis* Tul. Here, the presence of* 5,3*′*-hydroxy-7,4*′*-dimethoxyflavanone* in* P. gracilis* Tul. fruits and its antileishmanial activity* in vitro* and* in vivo* are reported for the first time.

## 2. Materials and Methods

### 2.1. General Experimental Procedures

^1^HNMR and ^13^C NMR spectra (all in CDCl_3_) were recorded on Bruker AMX 300 NMR spectrometers, using TMS as internal standard. Silica gel 60 (Merck, 0.063–0.200 mesh) was used for column chromatography, and precoated silica gel plates (Merck, 60 F_254_, 0.2 mm) were used for TLC.

### 2.2. Plant Material

Plant material was collected in the village of Santa Elena, “Sector Silletero,” municipality of Medellin, department of Antioquia (Colombia), in January 2011 at 2540 m.o.s.l. A voucher specimen was deposited in the Herbarium of the University of Antioquia (number 4588, F. Alzate).

### 2.3. Preparation of Extracts and Partial Purification

Material was dried in an oven at 35°C for 48 hours. Powdered leaves (86 g) of* P. gracilis* Tul. were extracted successively with hexane, then dichloromethane and ethyl acetate, and finally ethanol in a percolator at room temperature and concentrated in vacuum to give the corresponding extract (2.35 (2.7%), 5.63 g (6.5%), 4.59 g (5.3%), and 9.59 g (5.3%), resp.). On the other hand, 37.34 g of dried fruits was extracted with ethanol only and after concentration a dark brown paste was obtained, 7.88 g (21%). Presence of major compound was observed by TLC (mobile phase, dichloromethane). Then, extract was subjected to silica gel column chromatography eluting with a step gradient of* n-*hexane-ethylacetate (100 : 0, 90 : 10, 80 : 20, 70 : 30, 60 : 40, 50 : 50, 40 : 60, 30 : 70, 20 : 80, 10 : 90, and 0 : 100, each 200 mL) to obtain 20 fractions (F1–F20) collected on the basis of their TLC profiles. Fractions F4–F10 were recognized as the most interesting ones, due to the appearance of red spots after spraying with anisaldehyde reagent. Flavanone (350 mg, 4.4%) was isolated from this fraction by preparative TLC using* n*-hexane-ethyl acetate (4 : 1) mixture.

*5,3*′*-Hydroxy-7,4*′*-dimethoxyflavanone*. ^1^HNMR (CDCl_3_, 300 MHz): *δ* 2.83 (H-3*β*, dd, *J* = 3.1, 17.2 Hz), 3.12 (H-3*α*, dd, *J* = 12.9, 17.2 Hz), 3.85 (s, OCH_3_), 3.96 (s, OCH_3_), 5.37 (H-2, dd, *J* = 3.1, 12.9 Hz), 5.78 (OH), 6.09 (H-8, d, *J* = 2.3 Hz), 6.12 (H-6, d, *J* = 2.3 Hz), 6.92 (H-5′, d, *J* = 8.3 Hz), 6.98 (H-6′, dd, *J* = 1.9, 8.3 Hz), 7.09 (H-2′, d, *J* = 1.9 Hz); 12.07 (OH). ^13^C NMR (CDCl_3_, 75 MHz): *δ* 43.21 (C-3), 55.71 (OCH_3_), 56.07 (OCH_3_), 78.94 (C-2), 94.27 (C-8), 95.13 (C-6), 103.17 (C-10), 110.69 (C-5′), 112.70 (C-2′), 118.18 (C-6′), 131.56 (C-1′), 145.96 (C-3′), 147.03 (C-4′), 162.87 (C-9), 164.14 (C-5), 168.00 (C-7), 196.02 (C=O) (see Supplementary Data of the Supplementary Material available online at http://dx.doi.org/10.1155/2015/978379).

### 2.4. Studies* In Vitro* of Cytotoxicity

Cytotoxic activity of all extracts and pure compound was evaluated in the human U937 cell line by MTT method, as described previously [21, 22]. In brief, cells were cultured in RPMI 1640 medium supplemented with 10% fetal bovine serum (FBS), and 1% of antibiotics (penicillin-streptomycin (10.000 U/mL) at 100.000 cells/mL and six concentrations of each product (200, 100, 50, 25, 12.5, and 6.25 *μ*g/mL). Cells cultured in medium alone were used as negative control (no toxicity) while cells exposed to amphotericin B (AmB) were positive control (toxicity). Cells were incubated at 37°C, 5% CO_2_ for 72 hours; then, the effect of each product on the viability of cells was determined incubating exposed and unexposed cells for 3 hours with 10 *μ*L of* 3-(4,5-dimethylthiazol-2-yl)-2,5-diphenyltetrazolium bromide*. The MTT was reduced by succinate mitochondrial dehydrogenase to purple formazan that was then solubilized with 100 *μ*L/well isopropanol 50% and SDS 10% and its concentration was determined by optical density at 570 nm in a spectrometer (Benchmark BioRad). Each concentration of the product and unexposed cells was tested in triplicate in at least two different experiments.

The* in vitro *cytotoxicity was determined as the percentage of viability and growth inhibition obtained from the optical densities (O.D.) for each experimental condition using the formula: viability (%) = (O.D. treated cells/O.D. untreated cells) × 100, where O.D. of untreated cells correspond to 100% viability. In turn, growth inhibition (%) is calculated as 100 − % viability. Growth inhibition (%) data obtained for each experimental condition was used to calculate the lethal concentration 50 (LC_50_) by Probit analysis [23]. Compounds were classified using an arbitrary scale as follows: potentially toxic: LC_50_ < 100 *μ*g/mL; moderately toxic: LC_50_ > 100 and < 200 *μ*g/mL; and potentially nontoxic: LC_50_ > 200 *μ*g/mL.

### 2.5. Studies* In Vitro* of Antileishmanial Activity

The activity of each extract or metabolite obtained from* P. gracilis* Tul. was determined on Phorbol 12-myristate 13-acetate-differentiated U-937 cells infected with intracellular amastigotes of* L. (V.) panamensis *expressing the green fluorescent protein gene (MHOM/CO/87/UA140pIR-GFP) [24, 25]. One mL of cells was dispensed into each well of 24-well plate (300.000 cell/mL RPMI 1640 medium and 100 ng/mL). Plates were incubated at 37°C, 5% CO_2_ during 72 hours and then washed twice with phosphate buffer saline (PBS). U-937 cells were then infected with stationary phase promastigotes of* L. (V.) panamensis* in a proportion of 35 : 1 (parasites : cell). Plates were incubated for 3 hours at 34°C, 5% CO_2_ and after incubation cells were washed twice with PBS and incubated again for 24 hours at 37°C, 5% CO_2_. Infected cells were exposed to four serial concentration dilutions of each product (100, 25, 6.25, and 1.56 *μ*g/mL RPMI 1640 medium). In parallel, cells incubated in medium alone were used as control of infection (negative control) and cells exposed to AmB were used as control of leishmanicidal activity (positive control). After 72 hours of incubation at 37°C, 5% CO_2_ cells were removed using trypsin/EDTA solution and washed twice with PBS by centrifuging 10 min at 1100 rpm, 4°C. Then, cells were analyzed in an Argon laser flow cytometer (Cytomics FC 500MPL) by reading at 488 nm excitation and 525 nm emission. Ten thousand events were counted from each well. The percentage of infected cells was determined by dot plot analysis while the parasitic load was calculated by the mean fluorescence intensity using histogram analysis. Each concentration was assessed in triplicate in at least two independent experiments.

*In vitro *antileishmanial activity was determined as percentage of infection (viable parasites inside infected cells) according to the MFI units from flow cytometry analysis for each experimental condition using the formula: % infection = (FMI treated infected cells/FMI untreated infected cells) × 100, where FMI of untreated infected cells corresponds to 100% viable parasites. Then, % of reduction of infection was calculated using the formula: % inhibition = 100 − % infection. The % inhibition obtained for each experimental condition was used to calculate the effective concentration 50 (EC_50_) by Probit analysis [23]. Compounds were classified according to their antileishmanial activity using an arbitrary scale as follows: active: EC_50_ < 20 *μ*g/mL; moderately active: EC_50_ > 20 and <50 *μ*g/mL; or potentially nonactive: EC_50_ > 50 *μ*g/mL. The index of selectivity (IS) was calculated by correlating cytotoxicity with antileishmanial activity using the formula: IS = LC_50_/EC_50_.

### 2.6. Evaluation of Therapeutic Response and Toxicity of 5,3′-Hydroxy-7,4′-dimethoxyflavanone* In Vivo*

The therapeutic response of flavanone was tested in the hamster (*Mesocricetus auratus*) model for CL [26]. Briefly, previously anesthetized (ketamine 40 mg/kg and xylazine 5 mg/kg) hamsters were inoculated in the dorsal skin with promastigotes of* L. (V.) braziliensis* (MHOM/CO/88/UA301-EpiR-GFP) (5 × 10^8^ parasites/100 *μ*L PBS). Three experimental groups (*n* = 8 each), consisting of four males and four females, were coded accordingly: A: flavanone pure, B: 2% flavanone cream, and C: MA (positive control). Treatment with flavanone pure (40 *μ*L per dose), flavanone cream (40 mg per dose), or MA (200 *μ*g per dose) was initiated immediately after development of a typical ulcer (4–6 weeks after infection). Flavanone (groups A and B) was applied topically daily for 28 days. In turn, MA (20 *μ*L, 10 mg/mL) was applied intralesionally, also every day for 28 days. Animal welfare was supervised daily during the study. Both areas of the ulcer and body weight were measured every two weeks from the beginning of the treatments to the end of the study (three months after completion of treatment). The overall time points of evaluation were pretreatment day (TD0), end of treatment (PTD0), and posttreatment days 30, 60, and 90 (PTD30, PTD60, and PTD90, resp.). At the end of the study, hamsters were humanely sacrificed and, after necropsy, liver and kidney biopsies were taken for histopathological studies. A skin biopsy (from the site where the injury occurred) was also taken to determine parasite load by qPCR.

The effectiveness of each treatment was assessed comparing the lesion sizes prior to and after treatments. Treatment outcome at the end of study was recorded as* cure* (healing of 100% of the area and complete disappearance of the lesion);* clinical improvement* (reducing the size of the lesion in >30% of the area);* failure* (increasing the size of the lesion); or* relapse* (reactivation of lesion after initial cure). To compare the effectiveness among groups of treatments an arbitrary score was assigned to each treatment: 3 = cure, 2 = clinical improvement, 1 = relapse, and 0 = failure.

The toxicity of flavanone pure, flavanone cream, or MA was evaluated according to hepatic and renal functions of hamsters in treated and untreated animals as described previously [26]. At day TD0 and day 8 of treatment (TD8), blood was drawn from the heart and serum was separated by centrifugation at 5000 ×g for 2-3 min. The serum was stored at −80°C until use. Hepatic and renal functions were assessed by measuring the levels of alanine amino transferase (ALT), blood urea nitrogen (BUN), and creatinine using commercially available kits (Biosystems, Spain). The hepatic and renal functions were also evaluated in healthy (uninfected and untreated) hamsters. Toxicity of treatments was determined by comparing serum levels of ALT, BUN, and creatinine and postmortem histological changes in liver and kidney. Severity of histological changes was also graded as severe, moderate, or mild.

### 2.7. Quantification of Parasite Load

Quantification of parasites present in the lesions was based on amplification of a single copy gene of* Leishmania* DNA polymerase (housekeeping gene) using quantitative real time PCR (qPCR) and a standard curve as described elsewhere [27]. Initially, a 600 bp fragment of DNA of* L. (V.) panamensis* was amplified by conventional PCR (T1000 thermocycler, BioRad) and purified using QIAquick Gel Extraction Kit according to the manufacturer's instructions and product was ligated to InsTAclone pTZ57R/T vector. Then, DH5 alpha cells (Invitrogen, USA) were transformed with the construct. The construct was purified and, then, qRT-PCR was set to amplify a 120 bp fragment within the 600 bp sequence initially cloned. The number of copies per plasmid was determined based on the size of the cloned fragment and the size of the insert. A standard curve ranging from 1 to 1 million parasites in log increases from 10 was established. The QuantiFast SYBR Green qRT-PCR kit (Qiagen Inc., USA) was used for qPCR. The PCR amplifications were performed in a SmartCycler II (Cepheid, Sunnyvale, CA, USA) using a final volume of 25 *μ*L containing 100 ng of DNA, 12.5 *μ*L of the reaction mix, 100 nmol/L of each primer, and nuclease-free water. The amplification efficiency of each was measured using the PCR program LinReg. Tissue samples were weighted and lysed with 500 mL of lysis buffer (100 mM NaCl, 10 mM Tris-HCl, 25 mM EDTA, and 0.5% SDS, pH 8.0) and 0.1 mg/mL proteinase K by incubation in a water-bath for 4 hours at 56°C. Then, DNA was extracted with one mL of phenol : chloroform : isoamylic alcohol (25 : 24 : 1). After centrifugation for 10 min at 1700 rpm, the aqueous layer was carefully removed, washed with 90% ethanol, centrifuged 2 min at 500 ×g, and dried at room temperature. The pellet was resuspended in 300 *μ*L of autoclaved nuclease-free water. DNA was quantified using NanoDrop 1000 (Thermo Scientific, NH, USA) at 260 nm of absorbance and stored at −20°C until further use.

### 2.8. Data Analysis

For each parameter, average values with standard deviations (mean ± SD) were calculated. Data were analyzed by a two-way ANOVA. Differences were considered significant if *P* < 0.05. Statistical analysis was performed with Prism 6.0 (Graphpad Prism, San Diego, CA, USA).

## 3. Results 

**Figure 1:**
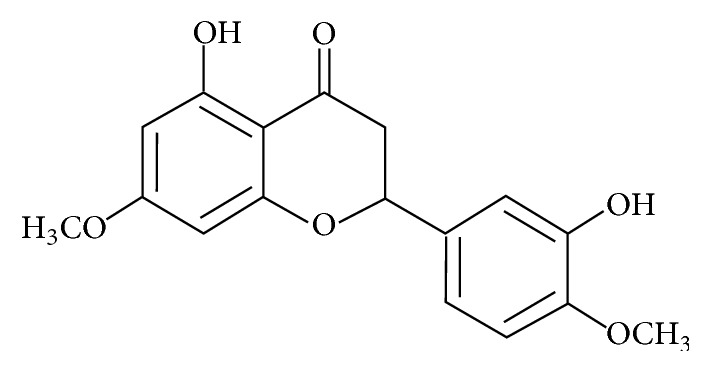
Chemical structure of* 5,3*′*-hydroxy-7,4*′*-dimethoxyflavanone* isolated from fruits of* Picramnia gracilis* Tul.

A majoritarian compound (4.4% yield) was identified as* 5,3*′*-hydroxy-7,4*′*-dimethoxyflavanone*, after NMR analysis and high resolution mass (Figure 1).

Ethanolic and ethyl acetate extracts from leaves (E-EtOH-l and E-EtAc-le) and ethanol extract from fruit (E-EtOH-fr) were potentially nontoxic for U-937 cells (LC_50_ > 200 *μ*g/mL) (Table 1) while hexane and dichloromethane extracts from leaves (E-He-le and E-DiClMe-le, resp.) were highly cytotoxic (LC_50_ 22.4 and 29.1 *μ*g/mL, resp., as shown in Table 1). AmB, which is a highly cytotoxic drug, showed a LC_50_ of 37.5 *μ*g/mL, while MA was nontoxic on U-937 (LC_50_ > 1000.0 *μ*g/mL). The* 5,3*′*-hydroxy-7,4*′*-dimethoxyflavanone* did not exhibit cytotoxicity on human macrophages (LC _50_ > 200 *μ*g/mL).

Data represent the mean value ± SD of cytotoxicity in terms of 50% lethal concentration (LC_50_) and leishmanicidal activity in terms of 50% effective concentration (EC_50_): E-EtOH-fr: ethanolic extract from fruit; E-EtOH-le: ethanolic extract from leaves; E-DiClMe-le: dichloromethane extract from leaves; E-He-le: hexane extract from leaves; E-EtAc-le: ethyl acetate extract from leaves; IS (index of selectivity) = LC_50_/EC_50_.

The E-EtOH-fr showed moderate leishmanicidal activity on intracellular amastigotes of* L. (V.) panamensis* (EC_50_ 35.7 *μ*g/mL) (Table 1), while E-EtOH-le and E-EtAc-le were nonactive against intracellular amastigotes of* L. (V.) panamensis* (EC_50_ > 100 *μ*g/mL). Unfortunately, E-He-le and E-DiClMe-le (EC_50_ > 22.4 and > 29.1 *μ*g/mL, resp.) had leishmanicidal activity at concentrations that are toxic to cells that are the host cells for* Leishmania* parasites. As expected, AmB and MA were highly active against intracellular amastigotes of* L. (V.) panamensis* (EC_50_ 0.06 and 6.8 *μ*g/mL, resp.). The* 5,3*′*-hydroxy-7,4*′*-dimethoxyflavanone* showed high antileishmanial activity (EC_50_ 17.0 *μ*g/mL, 53.7 *μ*M). This biological activity of* 5,3*′*-hydroxy-7,4*′*-dimethoxyflavanone* was highly selective with an IS > 11.8 while in extracts from fruit and leaves the IS was <2.0 (Table 1). AmB and MA had an IS of 625.0 and >147.1, respectively.

Treatment of* L. (V.) braziliensis *40 *μ*L/2 mg/kg body weight/day/for 28 days with flavanone (group A) resulted in clinical improvement in 4/6 hamsters with 40 to 80% of reduction in their lesion sizes, failure in 1/6 hamsters, and relapse in 1/6 hamsters. Parasite load in this group of animals was 392.3 + 192.7 parasites/mg of tissue. On the other hand, treatment with 40 mg/2% flavanone cream/day/for 28 days (group B) produced cure in 2/6 hamsters (with 100% reduction of lesion size); clinical improvement (>80% of reduction in their lesion size) was observed in 2/6 hamsters and relapse was in 2/8 hamsters (reactivation of ulcer after initial cure at PTD45 or PTD75). In this group parasite load was 2405.0 ± 4312.6 parasites/mg of tissue. Finally, treatment with intralesion injection of MA 200 *μ*g/day/twice a week/28 days (group C) cured 5/6 hamsters and 1/6 hamster experienced relapse, 15 days after treatment. The amount of parasites in hamsters cured was 218.5 ± 393.7 parasites per mg of tissue. The parasite load in hamsters treated with flavanone solution (group A) was similar to that detected in hamsters treated with MA (group C), while it was different from that detected in hamsters treated with flavanone cream (*P* < 0.005).

**Figure 2:**
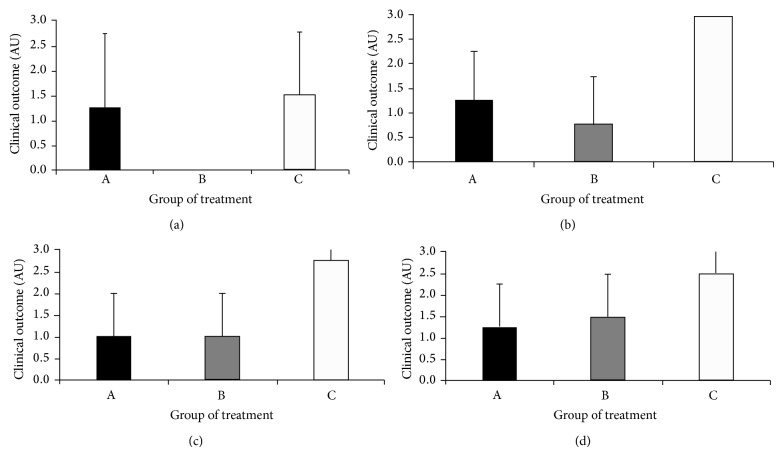
Clinical outcome after treatment with topical* 5,3*′*-hydroxy-7,4*′*-dimethoxyflavanone* isolated from* P. gracilis* Tul. fruit. Bars represent the mean value ± SD of clinical outcome in arbitrary units scored at the end of the treatment (a), PTD30 (b), PTD60 (c), and PTD90 (d). Axis *x*: group treated with flavanone solution (group A); group treated with 2% flavanone cream (group B); and group treated with trademark meglumine antimoniate (group C). Axis *y*: score of 0 = failure; 1 = relapse; 2 = clinical improvement; and 3 = cure.

Differences in treatment effectiveness were based on results obtained in each of the different stages of evaluation: end of treatment (PTD0) and PTD30, PTD60, and PTD90, using the arbitrary scale as described in the Materials and Methods. The mean value of treatment outcomes obtained at each time point for each treatment group is summarized in Figure 2. Effectiveness of topical treatment with topical flavanone pure (group A) was similar to that observed with intralesion injection of MA (group C) at any time point during follow-up except at PTD90 where response was higher in group C. Differences were not statistically significant (*P* > 0.05). On the other hand, the effectiveness of topical flavanone cream (group B) was higher than flavanone pure at PTD90. These differences were not statistically significant (*P* > 0.05).

Some animals in each treatment group experienced weight loss < 10%. Thus, loss weight was not associated with toxic effects of treatment with* 5,3*′*-hydroxy-7,4*′*-dimethoxyflavanone*. Levels of serum ALT for liver dysfunction and BUN and creatinine for renal dysfunction, measured 8 days upon treatment with this flavanone, as well as uninfected and untreated hamsters demonstrated increased levels of the serum of ALT and BUN after treatment with flavanone formulation, while creatinine levels were mildly increased after treatment with MA. However these levels were similar to those observed in hamsters infected but untreated (Table 2). Differences were not statistically significant (*P* > 0.05).

On the other hand, no histological alterations attributable to treatment were observed in animals treated with* 5,3*′*-hydroxy-7,4*′*-dimethoxyflavanone*. In contrast, hamsters treated with MA induced the following changes, which were observed in the liver: cloudiness, vacuolar and fat degeneration, karyomegaly, binucleation, and pigmentation. These occurred in moderate to severe degree. MA treatment also induced changes in kidney, including vacuolar and fat degeneration and binucleation in mild to moderate degree.

## 4. Discussion

Cutaneous leishmaniasis is an infectious disease that can cause serious psychologic and social stigma, especially when face and other visible areas of the body are compromised. Hereby, in the search of new or better drugs to treat CL, antileishmanial activity and cytotoxicity products derived from leaves and fruit of* P. gracilis* Tul. were tested* in vitro*. Only ethanolic extract of fruit had moderate leishmanicidal activity on intracellular amastigotes of* L. (V.) panamensis* and no cytotoxicity on macrophages and the antileishmanial activity and toxicity of the majoritarian compound of this extract was validated* in vivo*.

The majoritarian compound was identified as follows. This same flavanone was previously isolated from* Chromolaena odorata *(L.) (Asteraceae) [28],* Artemisia campestris *subsp*. maritima *(Asteraceae) [29], and* Heliotropium glutinosum* Phil. (Heliotropiceae) [30] and named* eriodictyol-7,4*′*-dimethyl ether*. However, the presence of* 5,3*′*-hydroxy-7,4*′*-dimethoxyflavanone* as a majoritarian compound in one species of the Picramniaceae family is reported for the first time. Additionally, activity of* 5,3*′*-hydroxy-7,4*′*-dimethoxyflavanone* against* L. (V.) panamensis *and* L. (V.) braziliensis *is reported also for the first time. Other flavanones have been reported having activities against* Leishmania* species and* T. cruzi*; thus, for example,* 5,6,7-trihydroxy-4-methoxyflavanone *isolated from methanol extract of* Baccharis retusa *(Asteraceae) showed activity against promastigotes of* L. (V.) braziliensis*,* L. (L.) amazonensis,* and* L. (L.) chagasi* (IC_50_ 49.0, 53.0, and 57.0 *μ*g/mL, resp.) and intracellular amastigotes of* L. (L.) chagasi* (IC_50_ 45.0 *μ*g/mL). This* 5,6,7-trihydroxy-4-methoxyflavanone *was also active on trypomastigotes of* T. cruzi* (IC_50_ 20.4 *μ*g/mL) without cytotoxicity to mouse peritoneal macrophages but with considerable toxicity to rhesus monkey kidney cells (LLC-MK2) and tumoral monocyte THP-1 cells (LC_50_ 31.0 and 49.0 *μ*g/mL, resp.) [31, 32]. Similarly,* 5,4*′*-dihydroxy-7-methoxyflavanone* (sakuranetin-2) isolated also from* B. retusa *was active against promastigotes of* L. (L.) amazonensis*,* L. (V.) braziliensis*,* L. (L.) major*, and* L. (L.) chagasi *(IC_50_ 51.9, 45.1, 52.6, and 38.4 *μ*g/mL, resp.). This flavanone was also active against intracellular amastigotes of* L. (L.) chagasi* with an IC_50_ value of 43.7 *μ*g/mL. No toxicity to Balb/c mice peritoneal macrophages was observed. However, this compound showed considerable toxicity to kidney cells LLC-MK2 and human monocytes THP-1 cells (IC_50_ 25.9 and 39.5 *μ*g/mL, resp.). This flavanone also was active on* T. cruzi* trypomastigotes (IC_50_ 20.2 *μ*g/mL) [33].

In addition,* 5,3*′*-hydroxy-7,4*′*-dimethoxyflavanone* reported here was active on intracellular amastigotes but inactive on axenic amastigotes of* L. (V.) panamensis *(data not shown). This result suggests that the flavanone may require metabolization after internalization by the host cell to produce the metabolite responsible for the leishmanicidal activity, as is seen with pentavalent antimony [33]. Although the possibility of metabolization by the host cell remains to be determined, antileishmanial activity of* 5,3*′*-hydroxy-7,4*′*-dimethoxyflavanone* on intracellular parasite may be related to its ability to chelate iron (Fe), depriving this essential nutrient from the intracellular forms [34].

The* 5,3*′*-hydroxy-7,4*′*-dimethoxyflavanone* administered at 2 mg/kg body weight/day (solution) or 2% (cream) during 28 days was able to induce cure or clinical improvement of CL in hamsters experimentally infected with* L. (V.) braziliensis*. Although effectiveness of flavanone was lower than MA in terms of treatment outcome at the end of the study, parasite load was similar in both groups of treatments. These results confirm that* 5,3*′*-hydroxy-7,4*′*-dimethoxyflavanone* is able to kill intracellular amastigotes of* L. (V.) braziliensis *present in the ulcer, but the efficiency is affected not only by dose but also by pharmaceutical formulation. None of the treatments produced detrimental effect on the body weight, histological morphology, or blood levels of ALT, BUN, and creatinine attributed to toxic effects of flavanone. Because the levels observed after treatment were similar to those in infected/untreated hamsters, variations are probably associated with* Leishmania *infection process. Moreover, nitrogen compounds may be increased due to the high degradation of amino acids or high-protein diets and are not always associated with an alteration in the kidney [35].

## 5. Conclusion

Hereby, the* 5,3*′*-hydroxy-7,4*′*-dimethoxyflavanone* is reported for the first time in one species of the Picramniaceae family. The activity against* L. (V.) panamensis *and* L. (V.) braziliensis *is also reported for the first time. Overall, bioassay testing results observed in this investigation indicate that* 5,3*′*-hydroxy-7,4*′*-dimethoxyflavanone* isolated from* P. gracilis *Tul. represents promising antiprotozoal leads for further development of drugs to treat CL. Therapeutic response of this flavanone would be improved by increasing the amount of the active ingredient in the formulation, increasing the frequency of administration or extending the days of treatment. Finally, this work contributes with new knowledge chemical composition and novel biological activity of* P. gracilis* Tul., a native species from Central and South America.

## Supplementary Material

Supplementary Material provides the NMR spectra (^1^HNMR and ^13^CNMR) of 5,3-hydroxy-7,4-dimethoxyflavanone.

## Figures and Tables

**Table 1:** *In vitro* cytotoxicity and antileishmanial activity of *Picramnia gracilis* Tul.

Product	Biological activity (*μ*g/mL)		IS
	LC_50_	EC_50_	
E-EtOH-le	>200.0	>100.0	>2
E-He-le	22.4 ± 5.4	>22.4	<1
E-DiCl-Me-le	29.1 ± 9.0	>29.1	<1
E-EtAc-le	>200.0	>100.0	>2
E-EtOH-fr	>200.0	35.7 ± 1.3	>2
*5,3*′*-Hydroxy-7,4*′*-dimethoxyflavanone*	>200.0	17.0 ± 2.8 (53.7 *μ*M)	>11.8
Amphotericin B	37.5 ± 7.6	0.06 ± 0.004	625.0
Meglumine antimoniate	>1000.0	6.8 + 0.5	>147.1

Data represent the mean value ± SD of cytotoxicity in terms of 50% lethal concentration (LC_50_) and leishmanicidal activity in terms of 50% effective concentration (EC_50_). E-EtOH-fr: ethanolic extract from fruit; E-EtOH-le: ethanolic extract from leaves; E-DiClMe-le: dichloromethane extract from leaves; E-He-le: hexane extract from leaves; and E-EtAc-le: ethyl acetate extract from leaves. IS (index of selectivity) = LC_50_/EC_50_.

**Table 2:** Blood levels of ALT, BUN, and creatinine in hamsters treated with *5,3*′*-hydroxy-7,4*′*-dimethoxyflavanone*.

Group	ALT (U/L)	BUN (mg/dL)	Creatinine (mg/dL)
(A) TD0			
Healthy (*n* = 5)	64.4 ± 5.6	15.9 ± 2.6	0.4 ± 0.1
Infected/untreated (*n* = 3)	73.9 ± 2.8	18.2 ± 1.9	0.4 ± 0.14
Flavanone solution (*n* = 6)	67.8 ± 3.2	18.0 ± 4.9	0.5 ± 0.1
Flavanone cream (*n* = 6)	65.8 ± 17.6	17.4 ± 1.3	0.3 ± 0.02
MA (i.l) (*n* = 6)	57.0 ± 6.1	20.9 ± 6.9	0.4 ± 0.1
(B) TD8			
Flavanone solution (*n* = 6)	76.8 ± 3.7	20.0 ± 2.2	0.4 ± 0.03
Flavanone cream (*n* = 6)	77.1 ± 7.6	22.9 ± 6.4	0.3 ± 0.1
MA (i.l) (*n* = 6)	62.0 ± 8.5	23.8 ± 2.2	0.8 ± 0.1

Data represent the mean values ± SD of *n* animals per group of treatment.

## Abbreviations

CL: Cutaneous leishmaniasis
LC_50_: Lethal concentration 50
EC_50_: Effective concentration 50
IS: Index of selectivity.

## References

[1] Alvar J., Vélez I. D., Bern C. (2012). Leishmaniasis worldwide and global estimates of its incidence. *PLoS ONE*.

[2] World Health Organisation (2010). Control of the leishmaniases. *WHO Technical Report Series: Report of a Meeting of the WHO Expert Committee on the Control of Leishmaniases*.

[3] Newman D. J., Cragg G. M. (2012). Natural products as sources of new drugs over the 30 years from 1981 to 2010. *Journal of Natural Products*.

[4] Fernando E. S., Quinn C. J. (1995). Picramniaceae, a new family, and a recircumscription of *Simaroubaceae*. *Taxon*.

[5] Cortadi A., Andriolo L., Campagna M. N. (2010). Morphoanatomy of leaves barks and wood of Argentinean Simaroubaceae *sensu latu*. Part I: *Alvaradoa subovata* Cronquist, *Picramnia parvifolia* Engl., Picramnia sellowii Planch. and *Castela coccinea* Griseb. *Boletin Latinoamericano y del Caribe de Plantas Medicinales y Aromaticas*.

[6] Jacobs H. (2003). Comparative phytochemistry of *Picramnia* and *Alvaradoa*, genera of the newly established family Picramniaceae. *Biochemical Systematics and Ecology*.

[7] Balderrama L., Braca A., Garcia E., Melgarejo M., Pizza C., De Tommasi N. (2001). Triterpenes and anthraquinones from *Picramnia sellowii* Planchon in Hook (Simaroubaceae). *Biochemical Systematics and Ecology*.

[8] Herz W., Santhanam P. S., Wahlberg I. (1972). 3-epi-betulinic acid, a new triterpenoid from *Picramnia pentandra*. *Phytochemistry*.

[9] Rodríguez-Gamboa T., Fernandes J. B., Rodrigues Filho E. (2001). Triterpene benzoates from the bark of *Picramnia teapensis* (Simaroubaceae). *Journal of the Brazilian Chemical Society*.

[10] Rodríguez-Gamboa T., Victor S. R., Fernandes J. B. (2000). Anthrone and oxanthrone C,O-diglycosides from *Picramnia teapensis*. *Phytochemistry*.

[11] Diaz F., Chai H.-B., Mi Q. (2004). Anthrone and oxanthrone c-glycosides from *Picramnia latifolia* collected in Peru. *Journal of Natural Products*.

[12] Rodríguez-Gamboa T., Fernandes J. B., Fo E. R., Das G. F. Da Silva M. F., Vieira P. C., Castro C. O. (1999). Two anthrones and one oxanthrone from *Picramnia teapensis*. *Phytochemistry*.

[13] Solis P. N., Ravelo A. G., Gonzalez A. G., Gupta M. P., Phillipson J. D. (1995). Bioactive anthraquinone glycosides from *Picramnia antidesma* ssp. fessonia. *Phytochemistry*.

[14] Hernández-Medel M. D. R., Pereda-Miranda R. (2002). Cytotoxic anthraquinone derivatives from *Picramnia antidesma*. *Planta Medica*.

[15] Hernandez-Medel M. D. R., Lopez-Marquez O., Santillan R., Trigos A. (1996). Mayoside, an oxanthrone from *Picramnia hirsuta*. *Phytochemistry*.

[16] Camacho M. D. R., Phillipson J. D., Croft S. L., Solis P. N., Marshall S. J., Ghazanfar S. A. (2003). Screening of plant extracts for antiprotozoal and cytotoxic activities. *Journal of Ethnopharmacology*.

[17] de Castro S. L., Pinto M. C., Pinto A. V. (1994). Screening of natural and synthetic drugs against *Trypanosoma cruzi*. 1. Establishing a structure/activity relationship. *Microbios*.

[18] Martínez M. L., Travaini M. L., Rodriguez M. V. (2013). Tripanocide and antibacterial activity of *Alvaradoa subovata* Cronquist extracts. *Boletin Latinoamericano y del Caribe de Plantas Medicinales y Aromaticas*.

[19] http://www.tropicos.org.

[20] Morton F. J. (1981). *Atlas of Medicinal Plants of Middle America—Bahamas to Yucatan*.

[21] Robledo S., Osorio E., Muñoz D. (2005). In vitro and in vivo cytotoxicities and antileishmanial activities of thymol and hemisynthetic derivatives. *Antimicrobial Agents and Chemotherapy*.

[22] Insuasty B., Ramírez J., Becerra D. (2015). An efficient synthesis of new caffeine-based chalcones, pyrazolines and pyrazolo[3,4-b][1,4]diazepines as potential antimalarial, antitrypanosomal and antileishmanial agents. *European Journal of Medicinal Chemistry*.

[23] Finney J. D. (1978). *Statistical Method in Biological Assay*.

[24] Pulido S. A., Muñoz D. L., Restrepo A. M. (2012). Improvement of the green fluorescent protein reporter system in *Leishmania* spp. for the *in vitro* and *in vivo* screening of antileishmanial drugs. *Acta Tropica*.

[25] Varela M. R. E., Muñoz D. L., Robledo S. M. (2009). *Leishmania (Viannia) panamensis*: an *in vitro* assay using the expression of GFP for screening of anti-leishmanial drug. *Experimental Parasitology*.

[26] Robledo S. M., Carrillo L. M., Daza A. (2012). Cutaneous leishmaniasis in the dorsal skin of hamsters: a useful model for the screening of antileishmanial drugs. *Journal of Visualized Experiments*.

[27] Carrillo-Bonilla L. M., Montoya A., Arbeláez N., Cadena H., Ramírez J., Robledo S. M. (2014). Migration of *Leishmania (Viannia) panamensis* and its persistence in healthy skin of hamster. *Revista U.D.C.A Atualidade y Divulgação Científica*.

[28] Ling S. K., Pisar M. M., Man S. (2007). Platelet-activating factor (PAF) receptor binding antagonist activity of the methanol extracts and isolated flavonoids from *Chromolaena odorata* (L.) King and Robinson. *Biological and Pharmaceutical Bulletin*.

[29] Vasconcelos J. M. J., Silva A. M. S., Cavaleiro J. A. S. (1998). Chromones and flavanones from *Artemisia campestris* subsp. Maritima. *Phytochemistry*.

[30] Modak B., Rojas M., Torres R., Rodilla J., Luebert F. (2007). Antioxidant activity of a new aromatic geranyl derivative of the resinous exudates from *Heliotropium glutinosum* Phil.. *Molecules*.

[31] Grecco S. S., Reimão J. Q., Tempone A. G. (2010). Isolation of an antileishmanial and antitrypanosomal flavanone from the leaves of *Baccharis retusa* DC. (Asteraceae). *Parasitology Research*.

[32] Grecco S. D. S., Reimão J. Q., Tempone A. G. (2012). In vitro antileishmanial and antitrypanosomal activities of flavanones from *Baccharis retusa* DC. (Asteraceae). *Experimental Parasitology*.

[33] Shaked-Mishant P., Ulrich N., Ephros M., Zilberstein D. (2001). Novel intracellular Sb-V reducing activity correlates with antimony susceptibility in *Leishmania donovani*. *The Journal of Biological Chemistry*.

[34] Sen G., Mukhopadhyay S., Ray M., Biswas T. (2008). Quercetin interferes with iron metabolism in *Leishmania donovani* and targets ribonucleotide reductase to exert leishmanicidal activity. *Journal of Antimicrobial Chemotherapy*.

[35] García D. E., Cova L. J., Briceño S. (2012). Metabolitos nitrogenados en el hámster dorado alimentado a base de harina de lombriz (*Eisenia* spp.). *Archivos de Zootecnia*.

