# Supplementary material for: Antileishmanial Effect of 5,3′-Hydroxy-7,4′-dimethoxyflavanone of Picramnia gracilis Tul. (Picramniaceae) Fruit: In Vitro and In Vivo Studies
Source: Adv Pharmacol Sci. 2015 Apr 30;2015:978379. doi: 10.1155/2015/978379 (PMC4430626; doi:10.1155/2015/978379)
Supplement: Supplementary file 1 — Supplementary Material provides the NMR spectra (1HNMR and 13CNMR) of 5,3-hydroxy-7,4-dimethoxyflavanone. [file 978379.f1.docx]

**Leishmanicidal activity of flavanone from the fruit of *Picramnia gracilis* Tul (Picramnaceae): studies *in vitro* and *in vivo***

Sara M Robledo ^a,*^ Wilson Cardona ^b^, Karen Ligardo ^a^, Jéssica Henao ^a^, Diana L Muñoz ^a^, Natalia Arbelaez ^a^, Andres Montoya ^a^, Fernando Alzate ^c^, Jairo Sáez ^b^, Juan M Pérez ^b^ and Victor Arango ^d^

^a^ PECET-Medical Research Institute, School of Medicine, University of Antioquia-UdeA. Calle 70 No. 52-21, A.A 1226, Medellín, Colombia.

^b^ Química de Plantas Colombianas, Institute of Chemistry, Exact and Natural Sciences School, University of Antioquia-UdeA; Calle 70 No. 52-21, A.A 1226, Medellín, Colombia.

^c^ Grupo de Estudios Botánicos, Institute of Biology, Exact and Natural Sciences School, University of Antioquia-UdeA, Calle 70 No. 52–21, A.A 1226, Medellín, Colombia.

^d^ Pharmacy School, University of Antioquia-UdeA; Calle 70 No. 52–21, A.A 1226Medellín, Colombia.

Corresponding Author

*Sara M. Robledo. PECET-Instituto Investigaciones Médicas, Facultad de Medicina, Universidad de Antioquia-UdeA. Calle 70 No. 52-21, Medellín, Colombia; e-mail: [sara.robledo@udea.edu.co](mailto:sara.robledo@udea.edu.co); Phone: (574) 219 6502

**^1^H and ^13^C NMR spectra**
